# Genomic sequencing of a dyslexia susceptibility haplotype encompassing *ROBO1*

**DOI:** 10.1186/s11689-016-9136-y

**Published:** 2016-01-27

**Authors:** Satu Massinen, Jingwen Wang, Krista Laivuori, Andrea Bieder, Isabel Tapia Paez, Hong Jiao, Juha Kere

**Affiliations:** Molecular Neurology Research Program, University of Helsinki, Helsinki, Finland; Folkhälsan Institute of Genetics, Biomedicum Helsinki, Helsinki, Finland; Department of Biosciences and Nutrition, and Science for Life Laboratory, Karolinska Institutet, Stockholm, Sweden

**Keywords:** Dyslexia, *ROBO1*, Whole genome sequencing

## Abstract

**Background:**

The *DYX5* locus for developmental dyslexia was mapped to chromosome 3 by linkage study of a large Finnish family, and later, roundabout guidance receptor 1 (*ROBO1*) was implicated as a candidate gene at *DYX5* with suppressed expression from the segregating rare haplotype. A functional magnetoencephalographic study of several family members revealed abnormal auditory processing of interaural interaction, supporting a defect in midline crossing of auditory pathways. In the current study, we have characterized genetic variation in the broad *ROBO1* gene region in the *DYX5*-linked family, aiming to identify variants that would increase our understanding of the altered expression of *ROBO1*.

**Methods:**

We have used a whole genome sequencing strategy on a pooled sample of 19 individuals in combination with two individually sequenced genomes. The discovered genetic variants were annotated and filtered. Subsequently, the most interesting variants were functionally tested using relevant methods, including electrophoretic mobility shift assay (EMSA), luciferase assay, and gene knockdown by lentiviral small hairpin RNA (shRNA) in lymphoblasts.

**Results:**

We found one novel intronic single nucleotide variant (SNV) and three novel intergenic SNVs in the broad region of *ROBO1* that were specific to the dyslexia susceptibility haplotype. Functional testing by EMSA did not support the binding of transcription factors to three of the SNVs, but one of the SNVs was bound by the LIM homeobox 2 (LHX2) protein, with increased binding affinity for the non-reference allele. Knockdown of *LHX2* in lymphoblast cell lines extracted from subjects from the *DYX5*-linked family showed decreasing expression of *ROBO1*, supporting the idea that *LHX2* regulates *ROBO1* also in human.

**Conclusions:**

The discovered variants may explain the segregation of dyslexia in this family, but the effect appears subtle in the experimental settings. Their impact on the developing human brain remains suggestive based on the association and subtle experimental support.

**Electronic supplementary material:**

The online version of this article (doi:10.1186/s11689-016-9136-y) contains supplementary material, which is available to authorized users.

## Background

Developmental dyslexia (DD), the most common learning disability, has a neurobiological and partially genetic etiology. More than 10 genes are considered as candidate susceptibility genes for DD based on genetic linkage studies, targeted association studies, chromosomal translocations, and chromosomal deletions. [1] Recently, also genome-wide association studies [2–4] as well as copy-number analysis [5, 6] have suggested novel genetic variants to confer susceptibility to DD.

**Fig. 1:**
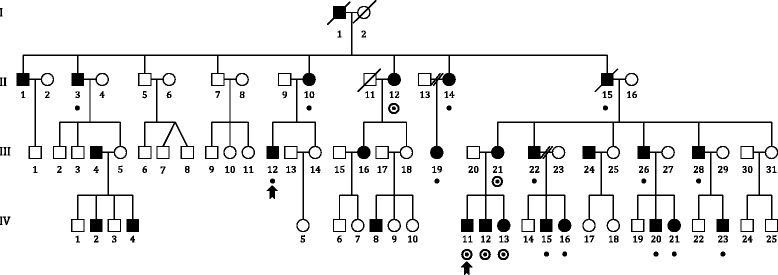
Pedigree of the *DYX5*-linked family. *Squares* denote males and *circles* females. The 19 affected individuals marked with *dots* share the dyslexia susceptibility haplotype [11], and their DNA samples were pooled for sequencing on the Illumina platform. The DNA from the two affected individuals denoted by *arrows* was used in the CGI WGS. *Circled dots* indicate the individuals whose DNA samples were used in the Sanger sequencing of the exonic SNPs

In the general population, DD mostly displays a complex occurrence pattern, although in some rare families Mendelian (monogenic) inheritance patterns have been observed. The largest family so far reported to show simple autosomal dominant segregation for DD is a Finnish family in which a genetic linkage study implicated a locus on chromosome 3, named *DYX5* [7] (Fig. 1), carrying a 33-Mb susceptibility haplotype for dyslexia. In neuropsychological tests, the affected family members had deficits in phonological awareness, verbal short-term memory, and rapid naming; most affected family members were classified as having severe DD while some were diagnosed with mild or compensated DD [8].

The *DYX5* locus was supported by a genome-wide scan where quantitative-trait loci (QTL) for DD were mapped in families from both the UK and USA [9]. Furthermore, in a QTL analysis on American families with speech–sound disorder (SSD), *DYX5* showed linkage to SSD-related phenotypes, suggesting that the locus may have pleiotropic effects [10].

The identity of the susceptibility gene in *DYX5* was fortuitously suggested by the chromosome translocation t(3;8)(p12;q11) in a dyslexic individual unrelated to the large family with *DYX5* segregation. The translocation breakpoint was fine-mapped to an intron of roundabout guidance receptor 1 (*ROBO1*) transcript variant 1 (NM_002941). Gene expression study of *ROBO1* in lymphoblasts from members of the large linkage family suggested suppressed expression from the rare haplotype segregating with DD. No such allelic suppression was observed for neighboring genes. [11] More recently, *ROBO1* was implicated by genetic association study in a core trait underpinning language acquisition, with a specific function in supporting a short-term buffer for arbitrary phonological strings [12]. An independent family-based analysis on Canadian samples provided more support for the association of *ROBO1* to DD, with the associated allele also correlating with low gene expression in brain tissue [13]. Consistent with an important developmental role of the *ROBO1* locus, a 15-Mb deletion involving *ROBO1* and a few neighboring genes was found in a child with developmental delay [14].

The *ROBO1* gene is orthologous to the roundabout axon guidance receptor regulating midline crossing of axons in fruit flies [15]. Homozygous *Robo1* knockout mice display a range of defects in axonal pathfinding, including anomalies in the development of the corpus callosum and other major axonal projections [16–18]. Although the affected members of the *DYX5*-linked family express *ROBO1* at reduced levels instead of lacking the expression completely, they also show a defect in axonal pathfinding, more specifically in the axonal crossing of auditory pathways. This was shown by using magnetoencephalography to record the cortical responses to frequency-tagged auditory stimuli; the ipsilateral suppression of auditory responses (which is dependent of midline crossing of the auditory pathways) was deficient in the dyslexic subjects who carried the dyslexia susceptibility haplotype. Moreover, the extent of this deficit in interaural interaction correlated with the expression level of *ROBO1* in lymphocytes in a dose-dependent manner [19].

The molecular mechanism for the suppressed expression of *ROBO1* from the DD susceptibility haplotype has remained unknown. The objective of this study was to characterize variation within the susceptibility haplotype in order to find variants that might shed light on the regulatory effects behind the dysregulation of *ROBO1*. We used a whole genome sequencing strategy to identify any rare variants that might affect gene regulation and tested the identified variants for function.

## Methods

### Sample selection

The DNA samples from 19 affected individuals sharing the same haplotype from the *DYX5*-linked family [7] were pooled (Fig. 1). The DNA concentrations were measured using Nanodrop 1000 spectrophotometer (Thermo Scientific), after which an equimolar pooled solution was prepared.

Out of the 19 individuals, DNA from 2 males, III.12 and IV.12 (Fig. 1), were also sent to full genome sequencing at Complete Genomics Inc. (CGI).

### Ethics, consent, and permissions

This study was approved by the ethical review board of the Helsinki University Central Hospital, and informed consent was obtained from the participants.

### Preparation of next-generation sequencing libraries and whole genome sequencing (WGS)

DNA libraries for sequencing were prepared using TruSeq DNA kits (Illumina Inc.) according to the manufacturer’s instructions with the following changes. The protocols were automated using an MBS 1200 pipetting station (Nordiag AB). All purification steps and gel-cuts were replaced by the magnetic bead clean-up methods as previously described [20, 21]. Whole genome sequencing of pooled affected individuals was performed on an Illumina HiSeq 2000 (Illumina Inc.) as paired-end reads to 100 bp. The clustering was performed on a cBot cluster generation system using a HiSeq paired-end read cluster generation kit (Illumina Inc.). The base conversion was done using OLB v1.9 (Illumina Inc.). Whole genome sequencing of two dyslexic male individuals was performed by CGI.

### Sequencing read analysis

Illumina sequence reads were aligned to National Center for Biotechnology Information (NCBI) human reference genome build 37, with Burrows-Wheeler Aligner (BWA) version 0.6.1 [22]. We set the threshold of base quality ≥20 for mapping. Genome Analysis Toolkit version 1.3 [23] was applied for insertion and deletion (INDEL) realignment and base recalibration, and we performed single nucleotide variant (SNV) and INDEL discovery in pooled samples using generalized ploidy model [24]. The reads with mapping quality less than 20 were not used for SNV and small INDEL calling. The CGI production pipeline mapped original sequence data to NCBI reference genome build 37 with Complete Genomics Analysis Tools (CGA™ Tools).

### Variant annotation and filtering

ANNOVAR software [25] was used for variant annotation and filtering. RefSeq, dbSNP 137 and data from 1000 Genomes Project (2012 February data release) [26], 1000 Genomes Project Finnish data, and Finnish population data from Sequencing Initiative Suomi (SISu) were used to annotate variants. Moreover, Integrative Genomics Viewer [27], Ensembl Genome Browser [28], and UCSC Genome Browser [29] including the Encyclopedia of DNA Elements (ENCODE) project data [30] were applied for read and variant visualization.

We filtered the single nucleotide variants based on the following criteria:

- Select variants within the genomic area of the dyslexia susceptibility haplotype between markers D3S3039 and D3S3045 on chromosome 3 (bases 73842243–106990161)
- Select variants with same genotype in both CGI samples
- Select variants shared in both CGI samples and Illumina pooled samples
- Select heterozygotes
- Filter out the variants with minor allele frequency of Finnish samples in the 1000 Genomes Project [26] over 5 %
- Include novel variants (i.e., variants not annotated in dbSNP and/or 1000 Genomes Project [26] and/or SISu)

INDEL filtration was similar to the strategy for single nucleotide variant, but we kept the INDEL in both Illumina pooled samples and one of the CGI samples. The identification of structural variation, including copy number variation, inversion, and translocation, was performed in the CGI sequencing dataset by CGA Tools. We also compared the distance between mapped read pairs with the average insert size of the genomic library from Illumina paired-end sequencing for structural variation detection in a pooled-sample dataset.

### Transcription factor binding prediction

We employed TRANSFAC Public database [31], JASPAR database [32], UniPROBE database [33], and P-Match software [34] to predict the transcription factor (TF) binding sites on the intergenic variants.

### Sanger sequencing

To re-sequence the previously reported exonic variants in *ROBO1* [11], the DNA samples from four dyslexic individuals used for mRNA expression measurement [11] and one affected sibling (II.12, III.21, IV.11, IV.12, and IV.13; Fig. 1) were selected. The DNA samples of two dyslexic individuals sent to CGI were used for insertion validation, as well as two non-dyslexic family members. DNA samples from all of the 19 dyslexic individuals who carry the dyslexia susceptibility haplotype were re-sequenced to confirm that the novel SNV at position 84674201 (SNV 4 in Table 3) was true and shared by all 19 individuals.

The primers for the amplification and sequencing reactions were designed using Primer3 [35]. All PCR assays were performed with standard reagent concentrations and temperature profiles. Sequencing was performed using dye-terminator chemistry and automated sequencers (Applied Biosystems). Primer sequences are available on request.

### Plasmids

The plasmid construct used for LIM homeobox 2 (LHX2)-V5 overexpression, pLenti6.2-LHX2-V5, was obtained from DNASU plasmid repository [36]. The GFP-V5 control plasmid, pLenti6/V5-DEST-GFP, was a kind gift from Dr. Päivi Ojala.

The luciferase constructs were based on the pGL3 promoter backbone (Promega). We cut the vector with the restriction enzymes NheI and XbaI and ligated the inserts with matching sticky ends using T4 DNA liga. The inserts contained 33 bp surrounding and including the SNV4 C and T alleles, and the additional bases needed to generate the sticky ends. The insert sequences are read from the minus strand of the reference sequence because also the *ROBO1* is encoded on that strand. The sequences of the inserts were:

C-allele forward: 5′-CGT TCT TAC AAA GTC CCG TTA ATT AAT ATT GGT GG-3′

C-allele reverse: 5′-CTA GCC ACC AAT ATT AAT TAA CGG GAC TTT GTA AGA ACG AGC T-3′

T-allele forward: 5′-CGT TCT TAC AAA GTC CCA TTA ATT AAT ATT GGT GG-3′

T-allele reverse: 5′-CTA GCC ACC AAT ATT AAT TAA TGG GAC TTT GTA AGA ACG AGC T-3′

Similarly, the 33-bp insert with the alternative alleles was also inserted into the vector in reverse orientation (with the sticky end generating extra bases switching sides of the insert). pGL3 promoter was used as a control in the luciferase assay.

In *LHX2* knockdown in lymphoblast cell lines, we used the following lentiviral small hairpin RNA (shRNA) vectors from the The RNAi Consortium shRNA Library (TRC-Hs1.0): TRCN0000013418, TRCN0000013419, TRCN0000013420, and TRCN0000013422. As a control vector, we used the scramble construct SHC002.

### Cell cultures

All cell lines were cultured at 37 °C in a humidified atmosphere of 5 % (*v*/*v*) CO_2_/air. Human Embryonic Kidney 293 cells (HEK293) were cultured in Dulbecco’s modified Eagle’s medium (DMEM), supplemented with GlutaMAX (GIBCO) and 10 % fetal bovine serum (FBS), 100 U/ml penicillin, and 100 μg/ml streptomycin.

EBV-transformed lymphoblast cell lines from the *DYX5*-linked family and controls were cultured in Roswell Park Memorial Institute (RPMI) 1640 medium, supplemented with 2 mM L-glutamine, 5–15 % FBS, and 50 μg/ml gentamicin.

### Nuclear cell lysates

We made nuclear cell lysates overexpressing LHX2-V5 or GFP-V5. HEK293 cell lines were grown on 6-well plates and transfected with 3.3 μg plasmid DNA per well using Fugene HD Transfection Reagent (Promega) according to the manufacturer’s instructions. Forty-eight hours after transfections, approximately 6 × 10^6^ cells in total were collected from transfection reactions combining multiple wells from the 6-well plates and the nuclear soluble proteins were extracted using NE-PER Nuclear and Cytoplasmic Extraction Reagents (Thermo Scientific) according to the manufacturer’s instructions. The overexpression of LHX2-V5 was confirmed on Western blots using V5 mouse monoclonal antibody (R960, Life Technologies) (data not shown). The protein concentration of the nuclear extracts was measured using Pierce BCA Protein Assay Kit (Thermo Scientific). The nuclear extracts from retinal pigment epithelium (RPE)-1 cells were prepared according to Pierce NE-PER Nuclear Protein Extraction Kit (Thermo Scientific).

### Electrophoretic mobility shift assay (EMSA)

The EMSA was performed using LightShift Chemiluminescent EMSA Kit (Thermo Scientific) according to the manufacturer’s instructions. The wild-type or mutant probes were 5′-end-labeled in forward strands with biotin (Additional file 1 Table S1). The binding reactions were carried out according to standard protocol, with 2.5–4 μg nuclear lysates per lane. Protein-free biotin-labeled probes were loaded as negative controls.

In order to examine if LHX2 antibodies would affect binding reactions, we added 4 + 4 μg of the LHX2 antibodies, (N-20, Santa Cruz Biotechnology, 19342x) and LHX2 Antibody (C-20, Santa Cruz Biotechnology, sc-19344x). The validity of the antibodies was confirmed on a Western blot using LHX2-V5 overexpressing cell extracts (data not shown). In the competition assays, an unlabeled “cold probe” (5′-GGT GAT CAG TAA TTG GCT TCT CCC-3′) was incubated with a 1000-fold molar excess for 15 min at room temperature before the addition of the biotinylated probes.

The EMSA was performed according to a standard protocol. The binding of the probes to nylon membrane was carried out using a standard 312-nm transilluminator for 15 min or Trans-Blot SD Semi-Dry electrophoretic transfer cell (Bio-Rad) at 20 V for 30 min.

### Luciferase assay

HEK293 cells were grown on 96-well plates and cotransfected with three plasmids: (1) 162 ng of pGL3 promoter with inserts or empty pGL3 promoter vector (Promega), (2) 6.5 ng of control Renilla vector pRL-TK (Promega), and (3) 162 ng of LHX2-V5 vector or GFP-V5 vector. The transfections were done using Fugene HD (Promega) transfection reagent according to the manufacturer’s instructions.

The cells were harvested 24 h after transfection, and the luciferase activity was determined using the Dual-Luciferase Reporter Assay System (Promega). All data were normalized to Renilla luciferase.

### Knockdown of *LHX2* in lymphoblast cell lines

We first screened four lentiviral shRNA constructs targeted against *LHX2*. The transduction protocol was as follows. The cells were seeded on 24-well plates, 1.5 × 10^5^ cells per plate in a volume of 250 μl. Transductions were performed on the same day using 500 μl virus particles (titre was about 7 pg/ml) per well and 8 μg/ml polybrene. The cells were then incubated in a cell incubator at 37 °C for 10 min, followed by a centrifugation at 2500 rpm for 30 min and incubation at 37 °C for 4 h. After the transduction, the media was changed into regular RPMI media supplemented with FCS and gentamicin. The cells were harvested after 24 or 72 h.

RNA extraction from the cells was done using RNeasy Plus mini kit (Qiagen), and cDNA synthesis was performed using TaqMan® Reverse Transcription Reagents (Thermo Fisher Scientific). Real-time PCR was done using the following TaqMan probes: *LHX2* (Hs00180351_m1), *ROBO1* (Hs01560560_m1), glyceraldehyde-3-phosphate dehydrogenase (*GAPDH*, 4310884E), and *18S ribosomal RNA* (*rRNA*, 4310893E) (Thermo Fisher Scientific). The data were analyzed using the comparative threshold cycle (Ct) method. The Ct values were normalized against the geometric mean of *GAPDH* and *18S rRNA*. The normalized Ct values (ΔCt) of the samples with scramble control shRNA were subtracted from the ΔCt of the shRNA constructs targeted against *LHX2*, resulting in ΔΔCt values. The fold change was 2^−ΔΔCt^.

## Results

### Sequence analysis

Because the dyslexia susceptibility haplotype is large (33 Mb) and the known variants in the *ROBO1* area were not unique to the *DYX5*-linked family [11], we used whole genome sequencing (WGS) to characterize all genetic variation in the genomic area within the susceptibility haplotype surrounding *ROBO1*. We combined individual- and pooled-sample sequencing strategies; we sequenced the whole genomes of two affected individuals at Complete Genomics Inc. (CGI) and used Illumina sequencing on a pooled sample including an equal amount of DNA from all the 19 affected members carrying the susceptibility haplotype. The pooling strategy allowed us to unambiguously reconstruct the susceptibility haplotype as it was shared by all samples in the pool, whereas the other haplotypes were different, coming from different parents.

In the whole genome sequencing of two individuals performed at CGI, 97 % of the genome was covered in both individuals and over 80 % of the genome contained at least 30-fold reads. The average read depths of two individual samples were 51-fold and 55-fold. Coverage and read depth of the *ROBO1* region from the CGI dataset was over 50-fold. We obtained about 1058 million reads from the WGS of pooled samples by using Illumina platform, and over 92 % of them could be mapped to human reference genome build 37. After filtering out the PCR duplicates, we had 598 million mapped reads covering 91 % of the human genome. Pooled-sample sequencing on Illumina covered 12 % of the human genome over 30-fold (Table 1), and the average read depth was 19-fold. The whole *ROBO1* gene region was covered in Illumina sequencing mapped reads with an average depth of 24-fold including over 99.9 % of the 1-Mb upstream region.

**Table 1:** Read depth comparison between the CGI and Illumina platforms

	CGI sample 1 (%)	CGI sample 2 (%)	Illumina pool (%)
Read depth ≥5×	99.30	99.20	90.10
Read depth ≥10×	98.30	98	85.66
Read depth ≥20×	94.70	93	51.22
Read depth ≥30×	88.40	84.40	12.06

### Variant identification and validation

The majority of human genetic variation that is involved in the cis-acting regulation of transcription is located within 1 kb of the transcription start site, although the regulatory variation can extend at least 1 Mb upstream and downstream from the transcription start site [37]. Therefore, we chose to focus our variant identification in the genomic area that contains the introns and exons of the *ROBO1* gene and 1 Mb upstream from the promoter. We searched for single nucleotide variants (SNVs), small insertions and deletions (INDELs), and large structural variations (SVs). The two affected individuals sequenced at CGI shared 619 SNVs in the area of the *ROBO1* gene and 883 in the segment 1 Mb upstream. From the Illumina pooled sequencing data, we found 1276 SNVs in *ROBO1* and 1714 SNVs within 1 Mb upstream of *ROBO1*. To exclude false positive results, we compared the variant datasets between the two platforms. There were in total 597 intronic SNVs, 1 5′UTR SNV, and 852 intergenic SNVs in the 1-Mb upstream region shared in both platforms (Table 2). We did not detect any SNVs in the *ROBO1* coding regions.

**Table 2:** SNVs in *ROBO1* and 1-Mb upstream region on two samples from CGI and Illumina pooled samples

Genomic function	Shared variants^a^	Shared heterozygotes	Novel SNVs^b^
Exonic	0	0	0
Intronic	597	290	1
5′UTR	1	0	0
Upstream (1 Mb)	852	374	3

^a^Variants showing in both Illumina pooled samples and two CGI individual samples

^b^Not annotated in dbSNP version 137

We excluded homozygous SNVs, because the Finnish *DYX5*-linked family shows an autosomal dominant inheritance pattern [7]. After filtering, 288 intronic heterozygous variants and 374 upstream intergenic heterozygous variants were further annotated according to the 1000 Genomes Project. We found 14 intronic and 20 upstream variants with minor allele frequency (MAF) less than or equal to 5 % in all populations. We filtered the data further by comparing to the Finnish samples in the 1000 Genomes Project. It appeared that many variants had a substantially higher MAF in the Finnish population, which we subsequently excluded as population-enriched variants. Combining the filtering and annotation information, there were finally one novel intronic SNV and two novel intergenic SNVs within 1 Mb upstream left for further investigation (Table 3).

**Table 3:** Four unknown single nucleotide variants in *ROBO1* upstream region on chromosome 3. The alternative allele fraction (AAF) estimate refers to the pooled sample of 19 dyslexic individuals

Code	Position	Ref	Alt	Depth	AAF	Gene region	Distance to *ROBO1* (bp)
SNV1	79667838	A	G	19	0.53	*ROBO1* intron	+149,221
SNV2	79911063	G	T	15	0.60	Intergenic	−94,004
SNV3	80013510	T	C	15	0.67	Intergenic	−196,451
SNV4	84674201	C	T	32	0.53	Intergenic	−4,857,142

The intronic SNV (SNV1) was located between the first non-coding exon and the first coding exon of the brain-specific *ROBO1a* (GenBank:NM_002941.3) splice variant, i.e., upstream of the *ROBO1b*  (GenBank:NM_133631.3) variant. The intergenic SNVs (SNV2, SNV3) were 94 and 196 kb upstream of the first *ROBO1a* exon. Several transcription factors (TFs) were predicted to bind to the sites containing the three novel SNVs, and the variants might affect the binding of the TFs. Moreover, SNV1 was located in an enhancer region according to the FANTOM5 promoterome atlas [38]. However, electrophoretic mobility shift assay (EMSA) analyses using RPE-1 cell nuclear extracts did not support TF binding to SNV1, SNV2, or SNV3 (data not shown).

Because regulatory regions can have long-range effects on gene promoters, even from distances over 1 Mb [39], and because SNVs that regulate gene expression have been shown to be enriched in the area covering 5 Mb from the transcription start site [40], we extended our search region to 5 Mb upstream of the *ROBO1* promoter region (Additional file 2 Table S2). We found a novel SNV (SNV4 in Table 3) 4.8 Mb upstream situated in a conserved regulatory element identified by comparing the genomes of 29 mammals [41]. We confirmed the variant in 19 affected individuals by Sanger sequencing.

We found two insertions in 3′UTR region of *ROBO1*, which are SNP rs35691197 and rs113692951, in agreement with a previous result [11]. Among 242 small INDELs in *ROBO1* intronic and 1-Mb upstream region, 4 deletions and 34 insertions were novel but they were located in mononucleotide or dinucleotide repeat regions. One- to 2-bp INDELs in a repeat region are unlikely to have functional consequences if they appear as typical microsatellite repeats. After comparison to the Finnish samples in the 1000 Genomes Project, no other variants appeared functionally interesting.

Both individually sequenced samples showed a long deletion (rs6147914) between *ROBO1* exons 3 and 4 and could be confirmed by junction sequences also in Illumina pooled samples. This 300-bp deletion is, however, a common structural variation in different populations and thus unlikely to associate with the suppressed expression of *ROBO1*. A 1600-bp deletion together with an inversion was located at 250 kb upstream of *ROBO1*. This deletion was found in many Finnish samples in the 1000 Genomes Project, and in addition, we observed an inversion linked with the deletion, which shows similar but not identical inversion patterns as other 1000 Genomes Project samples. We then extended the searching region to 5 Mb upstream and found a 457-bp inversion in 2.4 Mb upstream of *ROBO1* and 400 kb upstream of *GBE1*. There was no inversion with such size reported in public genomic variant databases, and the inversion is not located in any repeat region. We performed Sanger sequencing on the inversion region of both CGI samples, but were not able to validate the inversion in one of the samples (IV.12), and the other sample (III.12) showed a homozygous inversion and insertion pattern in this region. Thus, the inversion was unlikely to be part of the DD susceptibility haplotype.

Consistent with our observations of assumingly neutral SVs within the genomic area of *ROBO1*, many SVs within the locus can also be found in the Database of Genomic Variants (data not shown) [42]. Their role in DD is unclear because information on reading performance of the individuals is not available.

The 33-Mb dyslexia susceptibility haplotype contains 168 RefSeq genes. In order to study the possible effect of genes other than *ROBO1*, we searched the whole susceptibility haplotype for rare (MAF < 5 % in 1000 Genomes Project [26] or Exome Aggregation Consortium (ExAC), Cambridge, MA) heterozygous coding SNVs. We found two rare coding SNVs in zinc finger protein 717 (*ZNF717*) but found no rare coding SNVs for any other genes (Additional file 3 Table S3).

### Re-sequencing of exonic variants in *ROBO1*

In *ROBO1*, there was a discrepancy between the genotype acquired by whole genome sequencing and our earlier results [11]. Therefore, we performed Sanger sequencing in the exons in which SNVs have previously been reported: exon 12, exon 18 (NM_002941.2), and 3′UTR (NM_133631) [11]. We found 6923T>G (rs7616243) in one affected individual and insertion DIP6203-6205 (rs113692951) in five affected individuals, but all of the rest of the previously reported exonic SNVs (1741G>A, 2974C>A, 6227C>A, 6483T>A, 6651T>A) were homozygous for the reference allele, and thus, we were not able to verify them as true variants [11]. The insertion rs35691197 (not reported in the previous study [11]) was validated in five affected individuals by Sanger sequencing.

### *ROBO1* expression

**Fig. 2:**
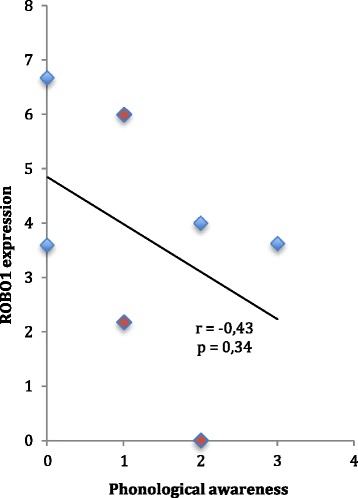
*ROBO1* expression and phenotype. The results from phonological coding tests were scored according to *z*-point comparisons to control group mean values, such that the larger the score, the more problems the subject had in phonological coding. The test scoring was 0 (*z* ≥ −1.0), 1 (−2.0 ≤ *z* < −1.0), 2 (−3.0 ≤ z < −2.0), or 3 (*z* < −3.0) [8]. The *ROBO1* gene expression was measured by real-time PCR from lymphocytes from the same subjects with higher values indicating higher expression [19]. *Blue diamonds* denote males and *red diamonds* females. The plotted values show a tendency for negative correlation, supporting the idea that the less *ROBO1* is expressed, the more deficit there is in phonological awareness

As our previous results for suppressed expression partially depended on the coding but unconfirmed variants, we proceeded to reconfirm if *ROBO1* gene expression correlates with the DD phenotype in the *DYX5*-linked family. We used the total gene expression values for *ROBO1* measured from lymphocytes [19] and compared them to the phenotypic test results of phonological awareness in the same individuals [8]. Overall, we confirmed that lower *ROBO1* expression correlated with more problems in phonological coding (Fig. 2).

### LHX2 binding site analysis

**Fig. 3:**
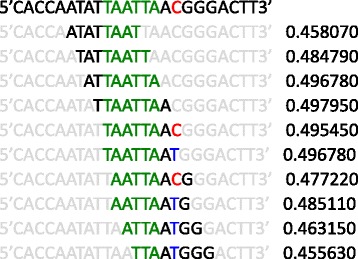
Predicted 8-mer binding sites for LHX2 in the genomic area of SNV4. The reference sequence (shown on the *top row*) was scanned for 8-mer binding profiles for transcription factors from the UniPROBE database [33]. The rows below the reference sequence show predicted 8-mer binding sites for LHX2, so that the bases that are not included in the 8-mer are shown in *grey*. The enrichment scores from the protein binding microarray data are shown rightmost on each row. The TAATTA consensus site for homeobox is shown in *green*, and the reference allele (cytosine) at SNV4 is shown in *red*. The alternative allele (thymidine) is shown in *blue*

SNV4 was situated in close proximity to a TAATTA element, which is a high-affinity binding site for homeobox transcription factors [43, 44]. LIM homeobox 2 (Lhx2) is a known negative regulator of the expression of *Robo1* in mice [45]. The genomic area of SNV4 was predicted to bind several transcription factors, including LHX2 in the UniPROBE database [33]. The T allele was predicted to create two more overlapping 8-mer binding sites for LHX2 [44] when compared to the C allele (Fig. 3), suggesting enhanced binding properties. Moreover, for those positions where there were predicted binding sites for both alleles, the enrichment scores indicating the binding affinity were slightly higher for the T allele. This led us to hypothesize that (1) LHX2 may bind to the genomic area of the SNV4 and that (2) LHX2 may have higher affinity for the T allele and possibly explain the suppressed transcription.

### Electrophoretic mobility shift assay

**Fig. 4:**
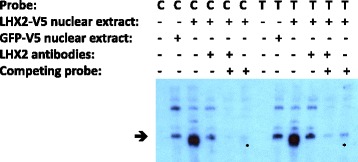
EMSA for SNV4. Nuclear extracts from HEK293 cells overexpressing LHX2-V5 or GFP-V5 were used. The *arrow* shows the location of the bands that showed differences in the amount of probe bound in a protein-DNA complex. A mixture of two antibodies against LHX2 was used to confirm that the protein-DNA complex at the level indicated by the *arrow* contains LHX2. An unlabeled probe was used to compete the binding of LHX2 to the SNV probes. The T allele seemed to be more resistant than the C allele to the competing probe as seen when comparing the bands above the *asterisks*

We tested the binding of LHX2 to the area of SNV4 by overexpressing LHX2 in HEK293 cell culture and using the nuclear protein extracts in EMSA. We tested the binding of LHX2 to our 33-bp probe including and surrounding SNV4. We detected enhanced binding affinity of our probes, the 33 bp including and surrounding SNV4 in the LHX2 overexpressing extracts compared to control extracts, confirming the binding of LHX2 to this site. Both of the allelic probes C and T bound LHX2 (Fig. 4). We also tested if we could alter the binding by adding antibodies that bind to LHX2. We did not detect a supershift, but instead, the binding of LHX2 to the probes was weakened when the antibodies were added, suggesting that the probes and the antibodies might compete in binding to the same site in the LHX2 protein and the addition of the antibodies hampered binding of the probe to LHX2. As a control, the addition of LHX2 antibodies to the green fluorescent protein (GFP) sample did not alter the binding of the probes (data not shown). Furthermore, we tested if we could compete the binding of the probe by LHX2 by adding an unlabeled cold probe containing a sequence from the CYP19A1 gene promoter that is known to bind LHX2 [46]. We observed that the cold probe did weaken the binding of our probe to LHX2 and that the SNV T was more resistant to the cold probe, suggesting that LHX2 may have higher affinity for the T probe than for the C probe. The binding of LHX2 was the least when both the competing probe and the LHX2 antibodies were added (Fig. 4). We obtained similar results in three independent EMSA assays.

### Luciferase assays

**Fig. 5:**
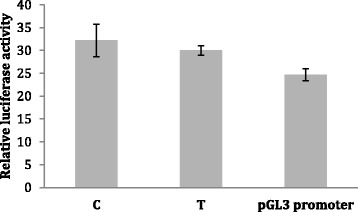
Luciferase assay for SNV4. The SNV4-containing pGL3-promoter vectors were tested for transcriptional activity in HEK293 cells by using luciferase assays. When combined as a group, the SNV4-containing vectors showed increased luciferase promoter activity when compared to the control empty vector (*P* < 0.05 on Student’s *t* test). We did not detect a significant difference between the C and G alleles. The *error bars* indicate standard deviation

We next studied whether SNV4 would bind to LHX2 also in cell line models. We cloned the same sequences that were used as probes in EMSA as inserts in luciferase vectors. In the LHX2 overexpressing cell lines, we were able to detect increased luciferase promoter activity of the insert containing vectors when compared to the control empty vector (*P* < 0.05 on Student’s *t* test; Fig. 5). This happened also when GFP was expressed as a control (data not shown). This may mean that there are some endogenous factors that bind to the inserts (similarly as there were protein-DNA complexes and the EMSA assays in the sample with GFP-V5 overexpression; Fig. 4). In this assay, we were not able to detect a difference between the SNVs (Fig. 5). Moreover, we also tested the inserts in reverse orientation, but this did not have a significant effect on luciferase expression (data not shown). The experiment was repeated three times with similar results.

### Knockdown of *LHX2* in lymphoblasts

**Fig. 6:**
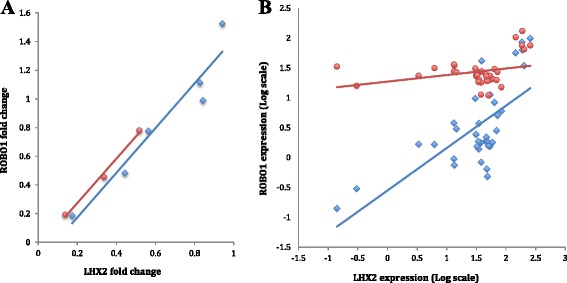
Correlation between *ROBO1* and *LHX2* expression. **a** *LHX2* was knocked down by lentiviral shRNA constructs in lymphoblast cell lines extracted from the *DYX5*-linked family and from control individuals. Expression levels of *LHX2* and *ROBO1* were measured by quantitative real-time PCR. The fold-change values indicate the difference between LHX2-shRNA and the control scramble shRNA-treated cells. *Blue* indicates the *DYX5*-linked family and *red* indicates controls. The *regression lines* show that low expression of *LHX2* correlates with low expression of *ROBO1*. **b** *ROBO1* and *LHX2* expression in 22 different brain tissues from the FANTOM5 database. The *red dots and lines* show the co-expression between *LHX2* and *ROBO1* shorter splice variant b. The *blue dots and lines* show the co-expression between LHX2 and *ROBO1* longer splice variant a

As Lhx2 has been shown to regulate *Robo1* in mice [45], we were interested to see whether the knockdown of human *LHX2* by using lentiviral shRNA vectors would affect *ROBO1* expression in lymphoblast cell lines. We first screened four constructs from the TCR1 library, and chose the one that gave the best reduction of *LHX2* expression levels measured by real-time PCR, which was on average 50 % of the *LHX2* expression levels in cells treated with scrambled shRNA. Next, we silenced *LHX2* in lymphoblast cell lines from individuals from the *DYX5*-linked family and control cell lines. We noted that *ROBO1* expression was reduced with downregulated *LHX2* expression, suggesting a regulatory effect of *LHX2* also in humans (Fig. 6a). We obtained similar results in two independent experiments.

### *ROBO1* and *LHX2* expression in the human brain

Our finding that *LHX2* knockdown in lymphoblast cells downregulated *ROBO1* expression was surprising in direction, as in mice Lhx2 has been found to be a negative regulator of *Robo1* [45]. Therefore, we used database information from the FANTOM5 database [47] to assess the expression pattern of *ROBO1* and *LHX2* in different human brain regions.

The correlation between *ROBO1* and *LHX2* in both fetal and adult human brain tissues indicated that the regulatory effect of *LHX2* on *ROBO1* expression may also be positive, suggesting cellular context-dependent complex regulation (Fig. 6b).

### Discussion

The combination of Illumina and CGI whole genome sequencing enabled us to characterize genetic variation in a large family with a weakly expressing haplotype of *ROBO1* gene and exclude variants unrelated to the shared haplotype. The average read depth of Illumina sequencing was 19-fold. If the pooled samples were distributed evenly, there was only a onefold coverage for each individual. Since we expected to identify a shared haplotype in all pooled samples, the low coverage per sample should not affect the detection of the susceptibility variants. However, at low sequencing depth, the SNV calling program would regard technical errors as variants and yield false positive results. Nevertheless, the CGI individual sequencing data, with over 50-fold average depth, compensated the weakness of low depth in Illumina sequencing. By comparing the variants between two individuals, we could exclude the variants with different genotypes and homozygous variants. After combining Illumina and CGI data, we excluded half of *ROBO1* SNVs detected from pooled samples. Those variants could be technical errors generated by Illumina platform or variants that were not shared by all affected individuals. There were variants found in CGI data but not detected by pooled sequencing. The lower coverage in pooled DNA sequencing is a potential risk factor for missing variants. However, some of them were located in well-covered regions, while the minor alleles only had less than 5× reads. Those variants might also only appear in those two individuals, but not in the shared haplotype linked to dyslexia. Technical errors in the CGI data interpretations might also mislead analyses. The filtering strategy requiring consistency across platforms greatly reduced the total number of candidate variants and might have resulted in filtering out true variants.

We used two different WGS sequencing platforms and Sanger sequencing but were unable to replicate previous findings on exonic SNVs [11], suggesting that they represent sequencing artifacts. Nevertheless, the hypothesis of the reduced expression was supported by the finding that *ROBO1* expression correlated with a measurable deficit in the crossing of auditory pathways in a dose-dependent manner [19] and that the lower expression of *ROBO1* associated with a more severe deficit in phonological coding (Fig. 2).

We found a new SNV at position 84674201 situated near a possible binding site for homeobox transcription factors (Fig. 3). We were able to show that the transcription factor LHX2, previously implicated in regulating *Robo1* in the mouse, has a higher affinity for the non-reference T allele. Also, luciferase experiments showed that transcription factors bind to both the reference C allele and the alternative T allele, but most probably, the luciferase assay was not sensitive enough to observe any differences between the two alleles. In the EMSA assay, the differences between the probes were detectable when a competing probe was used. We also found that when *LHX2* was knocked down in lymphoblast cell lines, there was a correlation between the expression of *ROBO1* and *LHX2*. In support of this finding and adding relevance to brain development, we observed that *ROBO1* and *LHX2* have a positive correlation in expression during human brain development, suggesting complex, possibly cellular context-dependent regulation.

In mice, Lhx2 is involved in the development of thalamocortical connections by regulating *Robo1* expression. Specific conditional deletion of Lhx2 in the thalamus alters projections from the medial geniculate nucleus [45]. This fits well with the previous findings of *ROBO1* regulating the crossing of the auditory pathways [19], because the medial geniculate nucleus is the area in the thalamus from which the auditory pathways connect to the auditory cortex [48]. Thus, altered *LHX2* binding is an attractive explanation for the etiology of dyslexia in the *DYX5*-linked family. Moreover, an unusual pattern of cell-size distribution within the medial geniculate nucleus [49] and abnormal thalamo-cortical connectivity have been observed in subjects with DD [50].

We characterized variation within a susceptibility haplotype for DD and found several novel variants, but at first glance, none of them stroke as a severe enough mutation to be a causative factor for DD with an apparent dominant effect in a large family. One of the pitfalls is the possible functional role of SNV1, SNV2, and SNV3. Our EMSA result that did not support the binding of nuclear factors to the common or variant sequences cannot be interpreted to exclude any functional effects in the developing brain. It is possible but less likely that we may have missed some variants despite our sequencing efforts, because the sequence coverage did not reach 100 %. It is also possible that we may have discarded a causative variant in our filtering steps that required consistency.

As a whole the 33-Mb dyslexia susceptibility haplotype contains roughly 1 % of the human genome, including 168 RefSeq genes. Interestingly, another member of the roundabout gene family, roundabout guidance receptor 2 (*ROBO2*), is located near *ROBO1* in a head-to-head orientation within the dyslexia susceptibility haplotype. Recently, a common variant near the 3′ end of *ROBO2* was associated with expressive vocabulary during the early phase of language acquisition [51]. Moreover, intronic deletions in *ROBO2* have been found in two independent cases with autism-spectrum disorders [52]. The *Robo1* and *Robo2* genes co-operate in axon guidance in mice during brain development [17] It is likely that also human *ROBO1* and *ROBO2* co-operate, and thus, *ROBO2* might contribute to the DD phenotype in the DYX5-linkage family. However, we did not find any novel coding variants within the *ROBO2* gene.

The expression pattern of *ROBO1* with multiple promoters and differential expression during development indicated that the regulation of *ROBO1* is likely to be complex. The experimental models available are most likely poor proxies of the molecular mechanisms during brain development, and in reality, very small effects on the experimental systems may well correspond to major effects during development. Therefore, any of the new variants discovered in the dyslexia susceptibility haplotype may be of functional relevance to explain the reduced expression of the *ROBO1* gene.

## Conclusions

We have characterized genetic variation in the area of a dyslexia susceptibility haplotype. Based on our data, despite the relatively large distance from the *ROBO1* promoter region, one SNV was implicated as a possible causal variant, even though the role of other discovered variants cannot be excluded.

## Additional files

Additional file 1: Table S1. The sequence pf the EMSA probes. (DOC 30 kb) Additional file 2: Table S2. Novel intergenic heterozygous SNVs detected by both platforms within 5 MB upstream of *ROBO1*. (DOC 43 kb) Additional file 3: Table S3. Rare coding heterozygous SNVs detected by both platforms in the linkage region. (DOC 28 kb)
