# Supplementary material for: Genomic sequencing of a dyslexia susceptibility haplotype encompassing ROBO1
Source: J Neurodev Disord. 2016 Jan 27;8:4. doi: 10.1186/s11689-016-9136-y (PMC4751651; doi:10.1186/s11689-016-9136-y)
Supplement: Additional file 1: Table S1. — The sequence pf the EMSA probes. (DOC 30 kb) [file 11689_2016_9136_MOESM1_ESM.doc]

**Supplementary table S1. The sequence pf the EMSA probes.**

| Probe | Sequence (5’-3’) |
| --- | --- |
| SNV1 A-allele | TTGCTCACAGAAAGCCTGTTTCATGGTCTC |
| SNV1 G-allele | TTGCTCACAGAAGGCCTGTTTCATGGTCTC |
| SNV2 G-allele | GAGATGATCTAGGGTATATGGCAGAAGAAT |
| SNV2 T-allele | GAGATGATCTAGGTTATATGGCAGAAGAAT |
| SNV3 T-allele | CAAAAGATAACAAGTGTTGGCAAGGATATG |
| SNV3 C-allele | CAAAAGATAACAAGCGTTGGCAAGGATATG |
| SNV4 C-allele | CACCAATATTAATTAACGGGACTTTGTAAGAAC |
| SNV4 T-allele | CACCAATATTAATTAATGGGACTTTGTAAGAAC |
