# Supplementary material for: Genomic sequencing of a dyslexia susceptibility haplotype encompassing ROBO1
Source: J Neurodev Disord. 2016 Jan 27;8:4. doi: 10.1186/s11689-016-9136-y (PMC4751651; doi:10.1186/s11689-016-9136-y)
Supplement: Additional file 2: Table S2. — Novel intergenic heterozygous SNVs detected by both platforms within 5 MB upstream of ROBO1. (DOC 43 kb) [file 11689_2016_9136_MOESM2_ESM.doc]

**Supplementary table S2. Novel intergenic heterozygous SNVs detected by both platforms within 5 MB upstream of *ROBO1***.

| chr | position | Ref | Alt | Gene Region | Distance to ROBO1 |
| --- | --- | --- | --- | --- | --- |
| 3 | 79911063 | G | T | intergenic | 94 004 |
| 3 | 80013510 | T | C | intergenic | 196 451 |
| 3 | 81106352 | G | C | intergenic | 1 289 293 |
| 3 | 81974369 | A | C | intergenic | 2 157 310 |
| 3 | 82939000 | C | T | intergenic | 3 121 941 |
| 3 | 82963250 | T | A | intergenic | 3 146 191 |
| 3 | 83014722 | C | T | intergenic | 3 197 663 |
| 3 | 83026186 | C | A | intergenic | 3 209 127 |
| 3 | 83908815 | T | C | intergenic | 4 091 756 |
| 3 | 84083724 | T | C | intergenic | 4 266 665 |
| 3 | 84298223 | G | A | intergenic | 4 481 164 |
| 3 | 84567561 | T | A | intergenic | 4 750 502 |
| 3 | 84604662 | C | T | intergenic | 4 787 603 |
| 3 | 84674169 | G | T | intergenic | 4 857 110 |
| 3 | 84674201 | C | T | intergenic | 4 857 142 |
