# Supplementary material for: Genomic sequencing of a dyslexia susceptibility haplotype encompassing ROBO1
Source: J Neurodev Disord. 2016 Jan 27;8:4. doi: 10.1186/s11689-016-9136-y (PMC4751651; doi:10.1186/s11689-016-9136-y)
Supplement: Additional file 3: Table S3. — Rare coding heterozygous SNVs detected by both platforms in the linkage region. (DOC 28 kb) [file 11689_2016_9136_MOESM3_ESM.doc]

**Supplementary table S3. Rare coding heterozygous SNVs detected by both platforms in the linkage region.**

| chr | position | Ref | Alt | Gene | Function | dbSNP |
| --- | --- | --- | --- | --- | --- | --- |
| chr3 | 75787725 | C | T | ZNF717 | nonsynonymous SNV | rs186706183 |
| chr3 | 75787729 | G | A | ZNF717 | nonsynonymous SNV |  |
